# Cross-National Differences in Psychosocial Factors of Perinatal Depression: A Systematic Review of India and Japan

**DOI:** 10.3390/healthcare5040091

**Published:** 2017-12-04

**Authors:** Mizuki Takegata, Yukiko Ohashi, Anisha Lazarus, Toshinori Kitamura

**Affiliations:** 1 Department of Pediatrics and Infectious Diseases, Institute of Tropical Medicine, Nagasaki University, Nagasaki 852-8523, Japan; 2 The Faculty of Human Science Technology, Bunkyo Gakuin University, Tokyo 113-8668, Japan; y-ohashi@bgu.ac.jp; 3 NPO Sangath, Goa 403501, India; anisha.lazarus@sangath.net; 4 Kitamura Institute of Mental Health Tokyo, Tokyo 151-0063, Japan; kitamura@institute-of-mental-health.jp; 5 Department of Psychiatry, Graduate School of Medicine, Nagoya University, Nagoya 466-8550, Japan

**Keywords:** cultural factors, India, Japan, perinatal depression, related factors, systematic review

## Abstract

Perinatal depression is prevalent worldwide. However, there are few available studies that discuss the different cultural factors affecting perinatal depression within Asian countries. This study aims to compare the literature regarding related factors relating to perinatal depression in India and Japan, and to synthesize the evidence common to both countries in addition to the country-specific evidence. We conducted a systematic review using several databases (CINAHL, MEDLINE, Pubmed, Ovid, SCOPUS, IndMED, and ICHUSI). Keywords were “antenatal depression” or “postpartum depression”, and “India” or “Japan”. Both Japanese and English language papers were reviewed. The identified evidence was compared between the two countries, as well as with non-Asian countries based on previous reports. In total, 15 articles on India and 35 on Japan were reviewed. Although several factors were shared between the two countries as well as with other non-Asian countries (vulnerable personality, being abused, age, marital conflict, and lower socio-demographic status), some differing factors were identified between India and Japan and non-Asian countries; India: poor socioeconomic status, living only with the husband, pregnancy not welcomed by the husband, a female baby, and poor relationship with in-laws; Japan: infertility treatment, conflict with work–life balance, poor relationships with biological mother or in-laws, and concerns about social relations with the other mother’s friends. To conclude, involving the family and community may be important for implementing both global standardized and culture-specific interventions. In India, treatment involving the in-laws may be effective because large family structure is a significant predictor of perinatal depression. In Japan, a family/community approach involving not only the mother’s family of origin but also the working environment is essential.

## 1. Introduction

Perinatal depression occurs during pregnancy and/or within the first 12 months after delivery [1]. Symptoms such as depressed mood, loss of interest or pleasure, decreased appetite, psychomotor agitation or retardation, fatigue, feelings of guilt, insomnia, and suicidal ideation occur in various combinations [2]. Around 6.5–12.9% of women have depressive symptoms antenatally and 19.2% have postnatal depression worldwide [1]. Perinatal depression is a major health concern because it deteriorates not only the quality of life of mothers, but also parenting, which negatively affects the mother–infant relationship and infant development [3]. Therefore, the early detection and management of perinatal depression is critical in community settings.

Perinatal depression is prevalent in both the developing and developed countries of Asia (3.5–63.3%) [4]. Only one review has synthesized the evidence regarding the factors related to postnatal depression in Asia. It cast light on (a) biological factors (e.g., anaemia, premenstrual cramps), (b) demographic factors (e.g., low income and primiparas), (c) interpersonal factors (e.g., intimate partner violence, unwanted pregnancy), (d) vulnerable personality (e.g., lower sense of coherence, lower self-esteem), (e) psychological factors (e.g., antenatal anxiety or depression), and (f) obstetric factors (e.g., Caesarean section and negative birth experiences) [4]. Klainin and Arthur’s study (2009) also mentioned (g) cultural factors (e.g., preferred male infant), which are specific to the Asian context [4]. This result suggests to us that a culturally adapted approach should be considered in Asia. However, in the previous review there may have been insufficient evidence to examine the cultural factors of each country in Asia, due to the limited amount of English papers on the subject [4]. Hence, expanding the review of the research to also include non-English papers is an important step towards gaining broad evidence which is more focused on the local community.

Additionally, selecting two countries in Asia and discriminating between the common worldwide [5,6] and country-specific factors would be helpful. Furthermore, it would also be useful to discuss the complex social and cultural background that underlies each country’s specific factors. In this review, we selected two countries: India and Japan. These countries were selected because their social and cultural aspects (such as family structures, fertility rate, and childrearing cultures) clearly differ from each other, although perinatal depression is prevalent in both India and Japan (India: antenatal depression, 6–26.3%; postnatal depression, 7.5–45.5%; Japan: antenatal depression, 5.6–5.8%; postnatal depression, 5.0–21.5%) [7,8,9,10]. India, located in South Asia, is the second most populous country in the world (over 1.2 billion people) with a total fertility rate of 2.4 [11]. India consists of multi-ethnic groups with diverse cultures and religions. India is also characterized by a joint family structure (63–86%), where male ancestors, unmarried female offspring, and the brides of male family members usually live together under the same roof. Women, during the perinatal period, are cared for by other family members in the same household [12,13]. In contrast, Japan, located in East Asia, faces a seriously low fertility rate (1.4 in 2014) and an increase in the older age population [11]. More women are likely to get married and deliver a baby at an older age. Most families are nuclear (60%), which has been a trend for the last three to four decades. There is a Japanese traditional custom called “Satogaeri bunben”: a woman at late-term moves back to her biological parents’ house for delivery in her home town, and is cared for until a few months postpartum [14]; women may migrate to their mothers’ homes for the delivery period.

This study aims to compare the literature regarding related factors affecting perinatal depression in India and Japan, and to synthesize the evidence common to both countries, and other non-Asian countries, in addition to country-specific evidence.

## 2. Methods

### 2.1. Search Strategies

Relevant papers were searched for using CINAHL, MEDLINE, Pubmed, Ovid, SCOPUS, IndMED (Indian articles), and ICHUSHI (Japanese articles). ICHUSI is an online database containing articles relevant to the health sciences, including medicine, dentistry, nursing, and veterinary, only inside of Japan. First, relevant published papers were searched for using the following search terms: “antenatal depression” or “postpartum depression”, and “India” or “Japan”. The search was restricted to articles from within the last 20 years (from the end of November, 1996, until the end of November, 2016). Related titles and abstracts were screened according to the inclusion criteria. We conducted full text screening (Indian literature: M.T. and A.L., Japanese literature: M.T. and Y.O.). At this stage, additional articles were retrieved using the reference lists of the already selected published journal articles. We assessed the quality of papers when deciding the final papers to be retained (Indian literature was assessed by M.T. and A.L., Japanese literature was assessed by M.T. and Y.O.). Although we conducted a quality assessment in reference to the ‘Strengthening the reporting of observational studies in epidemiology’ (STROBE) statement [15], papers which were acceptable according to the minimum criteria were included in this review in order not to miss smaller evidence.

### 2.2. Inclusion and Exclusion Criteria

Studies were included if they met the following criteria (see Table S1): (a) includes in the results the investigation of factors related to perinatal depression among Indian or Japanese mothers, (b) in the case of quantitative studies, the target population was community-dwelling mothers, (c) an observational study, (d) the study is conducted in India or Japan, (e) a quantitative or qualitative study, (f) is peer-reviewed original research, (g) an article which assured the minimum acceptable quality (clear description about design, enrollment number for each observational period, setting, measurements, conducted a statistical analysis, and approved by an ethical committee). In addition to these criteria, for Indian journals, only papers written in English were scrutinized. Although their official languages are English and Hindi (the languages spoken in India are numerous), English is more recommended and used in the academic field, from basic to higher education. Researchers and clinicians usually use English rather than Hindi when publishing papers. On the other hand, it is a fact that English is still a big barrier among Japanese researchers and clinicians. Japanese is used in all levels of education. There are many peer-reviewed journals that accept Japanese written articles in the mental health field. Hence, papers written in Japanese were included. Studies recruiting Indian or Japanese mothers who lived in other countries were excluded due to consideration of possible bias from a different cultural environment.

### 2.3. Review Process

Firstly, factors relating to perinatal depression were listed for India and Japan. Secondly, common factors and country-specific factors were extracted through discussion between M.T., Y.O., and T.K. by comparing evidence between India, Japan, and an updated literature review regarding perinatal depression worldwide [5,6].

## 3. Results

### 3.1. Article Extraction

**Figure 1:**
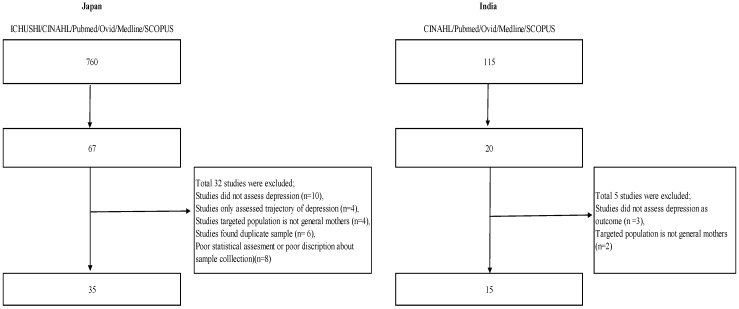
Related factors on perinatal depression systematic review: Flow diagram.

A total of 115 Indian and 756 Japanese papers were extracted from the search strategy (see Figure 1). Out of these, text screening yielded 20 (17%) papers on India and 67 papers (8%) on Japan. Finally, 15 papers (13%) on India [10,13,16,17,18,19,20,21,22,23,24,25,26,27,28] and 35 papers (4%) on Japan [7,8,9,14,29,30,31,32,33,34,35,36,37,38,39,40,41,42,43,44,45,46,47,48,49,50,51,52,53,54,55,56,57,58,59] were included in the review. Thirty-seven papers (India, *n* = 5; Japan, *n* = 32) were excluded because they did not fulfill the inclusion criteria: (a) three Indian and ten Japanese studies did not assess depression, but assessed other psychological aspects such as general health; (b) two Indian and four Japanese studies targeted a population of non-community-dwelling mothers; (c) four Japanese studies only assessed the trajectory of depression through the antenatal and postnatal periods without investigating related factors; (d) four Japanese studies did not target healthy mothers, (e) six Japanese studies were duplicates; and (f) eight Japanese studies were of poor statistical quality (unclear description of sample collection, poor sample calculation).

### 3.2. Prevalence and Common Factors

Out of 15 studies in India, 11 (73%) were of cross-sectional design (article numbers: I1–I6, I8–I12). Three studies assessed factors relating to antenatal depression (I5, I8, I12) and others investigated those for postpartum depression. The self-questionnaires used for assessing depression in India included the Edinburgh Postnatal Depression Scale (EPDS) (I1, I3–7, I10, I11, I13, I14) [60], the Patient Health Questionnaire-9 (PHQ-9) (I2) [61], the Kessler Psychological Distress Scale (K10) (I8, I9) [62], and the Beck Depression Inventory (BDI) (I12) [63]. The Clinical Interview Schedule-Revised (CIS-R) was also used in one study (I15) [64]. Twelve out of a total of 35 Japanese studies (34%) were of cross-sectional design (J5, J8, J9, J13, J17–19, J22, J24, J31–33). Eleven studies (31%) investigated antenatal depression (J1, J4, J5, J6, J8, J9, J13, J17, J21, J25, J30, J34). The self-questionnaires used for assessing depression in Japan included the EPDS (J2–4, J6–8, J10–16, J18–27, J31–35), the Zung Self-Rating Depression Scale (SDS) (J28, J29, J34) [65], the Hospital Anxiety and Depression Scale (HADS) (J1) [66], and the Center for Epidemiologic Studies Depression Scale (CES-D) (J5, J9, J17) [67]. Only one study assessed depression by means of Structured Clinical Interview for DSM (SCID-DSM-IV) (J30) [68].

Among the studies using self-rating questionnaires, the prevalence of antenatal depression ranged between 16–33% in India and between 6–41% in Japan, whereas that of postpartum depression is between 7–65% in India and between 8–29% in Japan. Using structured interviews, antenatal and postnatal depression prevalence was identified as 16 and 19%, respectively, in India; in Japan, a prevalence of 5% was identified for both antenatal and postnatal depressions.

### 3.3. Common Factors

Factors relating to perinatal depression identified across different countries were extracted through comparing the evidence between India, Japan, and non-Asian countries [5,6]. Associated factors of antenatal depression seen in India and Japan include demographic factors such as primiparas (J34), a history of mental disorder in the mother or family (I5, J4), younger age (J30), financial burden (J9, J25), and unwanted pregnancy (J9, J21, J30); vulnerable personality characteristics such as low self-directedness, high harm avoidance, and lower sense of coherence (J8, J13, J17); and interpersonal conflict including domestic violence (J1), experience of abuse (I12), and poor support from the husband or others (J13, J17).

Many factors associated with postnatal depression seen in the two countries, as well as other countries, overlapped with those of antenatal depression. They included demographic factors such as a history of mental disorder in the mother or family (I11, J27), younger age (I2, I9, I11, J14, J27, J18), and financial burden (I2, I4, I10, I13, I15, J2, J7, J9, J14); vulnerable personality characteristics such as a lower sense of coherence, lower self-esteem, neuroticism, and emotion-oriented coping styles (I6, J6, J10,J11, J17, J20, J23); interpersonal conflict such as domestic violence (I7), unplanned/unwanted pregnancy (I14, J9, J14, J21, J23), and poor support from husband or others (I15, J2, J3, J10, J14, J17, J18, J24, J29); and a negative life event (I15, J17). Other relevant factors which are characteristic of postnatal depression are psychological factors such as antenatal depression or anxiety (I6, J3, J12, J16), maternity blues (J26, J34), and a negative birth experience (J33).

### 3.4. Factors Identified Differently between the Two Countries

There was no factor specifically identified in each country as relating to antenatal depression. In studies in India (Table 1), poor socioeconomic status (below the poverty line, I2, I15), living only with the husband (I6), pregnancy not welcomed by the husband (I9), delivery of female baby (I1, I10, I11, I15), and poor relationships with in-laws (I15) were identified as correlating with postnatal depression. In contrast, we found the following factors correlate to postnatal depression in Japan (Table 2): infertility treatment (J21), conflict with work–life balance (J19, J23, J29), extended family household (J29, J31), poor relationships with biological parents or mother (J29, J32), concern about other relatives and in-laws (J14), and concern about social relationships with the other mother’s friends (J14, J24).

## 4. Discussion

This literature review compared evidence regarding perinatal depression in India and Japan. As well as common factors, some country-specific factors were identified. Whereas interpersonal factors relating to family characteristics strongly influence the onset of perinatal depression in Indian women, conflict within the work environment as well as with family members was a serious issue in Japanese women.

### 4.1. The Prevalence of Antenatal and Postnatal Depression

The prevalence in India ranged more widely (antenatal depression, 16–33%; postnatal depression, 7–65%) than other low–middle income countries [4,69,70]: many studies showed a prevalence of more than 20%, while Malaysia and Nepal had lower prevalences (4%) and Pakistan and Turkey had higher prevalences (Pakistan, 36–63%; Turkey, 14–50%). This may reflect the diverse cultures, settings, and ethnicites in India. The rate in Japan (antenatal depression, 6–41%; postnatal depression, 8–29%) is similar to that of other high-income countries [1,4,5]. Antenatal depression rates might be higher than other countries. However, the generalizability of the findings should be treated cautiously because it may be partly due to the various adopted cutoff points between the two countries and/or scales of depression used [60,61,62,63,64,65,66,67,68].

It is of note that most studies did not use a structured diagnostic interview such as SCID. Their diagnosis mainly relied on the results of questionnaires such as the EPDS. Cut-off points for the EPDS varied (11/12 vs. 12/13) depending on researchers. Some studies included in our review followed Patel’s study [10] using a cut-off score of 11/12. Although Fernandes et al. (2011), recommended cut off score of 12/13 in assessing antenatal depression in the rural setting [71], some studies assessed both antenatal/postnatal depression using the same cut off score. In Japan, there exists only one study examining the appropriate cut-off point for the EPDS using a combination of cases of major and minor depression [72]. This study proposed 8/9 as the best cut-off point for the EPDS to identify cases with major or minor depression. We must pay attention to the varying nature of appropriate cut-off points of questionnaires used over the perinatal period [73].

### 4.2. Common Factors

Several factors were associated with both antenatal and postnatal depression. Interpersonal conflict with partner—which includes domestic violence, experience of abuse, unwanted pregnancy, and poor support—is a major contributing factor to perinatal depression. Perinatal depression is a transitional period for both the husband and wife, which occurs as they gain parenthood, reform their relationship, adapt to a new environment, and welcome new family members. A crisis is more likely to occur in the process of transformation. In addition to this, being exposed to either traumatic events or abuse by the partner lowers an individual’s coping ability, which leads to being vulnerable to stressful events. While these factors linked to the onset of depression are probably universal, the results suggest that healthcare providers should be aware of both pregnancy planning and marital relationship-related factors when providing care.

### 4.3. Interpersonal Characteristics in the Cultural Contexts of Asian Countries

As several studies in this review demonstrated significant associations between having poor relationships with family, relatives, and others (e.g., mother’s friends, colleagues) and the onset of depressive symptoms during the perinatal period both in India and Japan, interpersonal relationships may be a key determinant in Asian societies. Whereas “individualism” is more valued in Western countries, “interdependence” is often described as an important value system in Asian cultures [74]. Kitayama et al. (1997), noted that “individuals (originally from western countries) are motivated to discover and develop positively valued internal attributes of the self, express them in public, and may develop a variety of social psychological processes that enable them to maintain and increase their self-esteem” [74]. This suggests that the satisfaction and well being of individuals are derived from having personal control over stressful situations in western countries. On the other hand, individuals in Asian countries are more required to adjust themselves into the society (organization). The spirit of cooperation, affection, and self-sacrifice, which are regarded as social virtues, are developed in individuals who grow through interactions with family, the work-place, and community members. Such virtues would be helpful to strengthen ties in family and work environments and are a great resource for mothers. However, we assume some women tend not to express their own needs and ask for help (self-sacrifice) because they recognize they should play their role (as a wife, as a colleague, and as a caregiver) adjusted to their family, work-place, and community (cooperation). Considering the the antenatal and postnatal period is challenging to mothers because it is also a dynamic period of transition to motherhood, the psychological distress of mothers within interpersonal relationships may be increased.

### 4.4. Interpersonal Factors in India and Japan

Although interpersonal factors such as poor relationships with family members and others are commonly identified in the two countries, social and cultural backgrounds may differ. Compared with Japan, India is characterized by strong ties within family members. A clear-cut gender role attitude exists in Indian society, where men work outside the home and women are responsible for doing household chores and caring for babies [75]. This may have pros and cons. Living in a large family, women are able to have more opportunities to share their experiences during the perinatal period. They can gain knowledge or skills for childrearing from other family members. This is also a source of economic security. There is also strong merit in mothers receiving assurance in several decisions regarding childrearing through consultations with family members. Having a good relationship with female family members would be important for the adaptive transition to motherhood. On the other hand, negative relationships between family members are likely to have adverse effects on the process of pregnancy, childbirth, and the postnatal period. Too much of an unequal power balance between the mother-in-law and bride (conflict with mother-in-law) results in little opportunity for the bride to express her feelings, leading to the onset of depression. If a woman lives only with her husband, it may be perceived as a serious lack of social support. Similarly, the situation where the woman is the sole decision maker about pregnancy and delivery is extremely stressful for an Indian woman.

In terms of Japan, the factor ‘extended family structure’ seems to be similar to the factor ‘poor relationship with mother-in-law’ identified in India, however, we assume these are different situations. According to Ninagawa [56], it was assumed that because of the increased workload (including house-chores), there may increased stress for mothers living in large families. The fact that ‘conflict with biological mother’ was identified as a significant contributor to perinatal depression is of interest as this may reflect a modern Japanese society where the majority of families are nuclear and relationships with other relatives are relatively scarce [76]. More mothers expect to receive emotional and practical support from their biological mothers as well as husbands. Regardless of ‘Satogaeri bunben’, biological mothers are key persons from whom to inherit related knowledge and skills regarding delivery and childrearing in the Japanese tradition [49,77]. However, there are some conflicts that occur between biological mothers and daughters that arise from differences between the traditional and modern childrearing styles.

It is of interest that mothers who were concerned about establishing social relationships with other mothers during pregnancy and after childbirth were more likely to manifest depression after delivery [40]. Pessimistic images regarding getting involved in new social relationships with other “mother-friends” after childbirth are a common topic of television programs or magazines. Nakayama (2013) noted that many mothers experienced conflicts with other mothers regarding different aspects: different opinions regarding parenting practice (e.g., the child of my friend is not disciplined), concerns about the difference in socioeconomic status, and hassles such as “secrets” being leaked to others [78]. Hence, Japanese mothers, sensitively seeking a community network, regard starting new relationships with other mothers in the community as a big concern.

In dual-income families in Japan, work–life conflict is significantly associated with perinatal depression. Japanese society has not caught up with the rapidly increasing number of working mothers [79]. This is reflected by the shortage of nursery rooms, long working hours, and an unfriendly atmosphere in offices regarding both the mother and husband being able to take a vacation for childrearing. Husbands cannot take sufficient time to spend with their family due to an uncooperative working environment. It is still very rare for a husband to take parental leave from an office or company in Japan. Another factor is that husbands are not used to doing household chores because their generation grew up with stay-at-home mothers due to traditional gender roles.

### 4.5. Preference of Male Infants, and Lower Socio-Economic Status in India

As well as in China, Taiwan, Hong-Kong, Korea, and Turkey [80], in India boys are preferred to girls because boys can take over the family business as a leader, contributing to the economic prosperity of the family. In contrast, girls are often considered as a financial burden under a custom called the “Dowry system”: where the family of bride should give a huge amount of money to the groom’s family when they marry, because the bride is supposed to belong to groom’s family and be financially supported after marriage. The dowry system is thought to put a great financial burden on the bride’s family. In some cases, the dowry system leads to female infanticide in India [27]. In terms of gender preference, Japan is exceptional among Asian countries in that there is not such a preference for boys. Male preference is found to have gradually weakened over the past two decades, and instead, female preference is becoming more dominant. Moriizumi (2008) found that the majority of couples had a balanced gender preference towards daughters and sons [81]. A possible reason for daughter preference is that parents are expecting biological daughters to take care of them when they become older, rather than financial support.

It should be noted that lower socio-economic status in India is much more influential in perinatal depression than in Japan. India has achieved strong economic growth (Gross Domestic Product = $2.26 trillion) contributing to a reduction in the poverty rate [82]. However, there is a big socio-economic gap that exists in some regions and social hierarchies called caste, which are regarded as a strong determinant of health in India, are present [83]. It is reported people belonging to scheduled castes (or scheduled tribes) are more likely to suffer from moderate/severe depression than others [84]. Social disadvantages such as lower education, poverty, and discrimination from others not only cause mental impairment, but also become an obstacle in access to care and treatment.

### 4.6. Implications for Clinical Practice and Suggestions for Future Study

Consistent with the previous literature review [4], many common factors with other countries were identified from this current review. There is ample literature on the psychological interventions for and prevention of perinatal depression [85,86,87,88]. Most of the reports were, however, from western countries. There has been a movement of Global Mental Health (GMH) to scale up evidence-based interventions in low–middle income countries, however, we should exercise caution when applying ‘universal’ prevention and interventions in culturally-different countries [89,90]. Socio-economic determinants regarding mental health may play a different role depending on each country. According to a previous study, which explored cross-country differences in the effects of socio-economic characteristics of patients with diabetes on subjective health-related wellbeing, clear differences were revealed in 15 countries [91]. Assari (2014) concluded each country should consider ‘the context that shapes social and behavioral determinants’ [91]. Considering these country-specific factors in our study, cultural adaptation of interventions or developing other culture-specific strategies is essential. Especially in India, enhancing social awareness of the importance of perinatal depression in family and community settings may be worth considering as a means of detecting at-risk populations for perinatal depression. Involving family members in a session is a well-known technique in interpersonal therapy [88] but this may be even more important in the prevention and treatment of perinatal depression in India. Involving not only the women themselves, but also family members such as mothers-in-law, in psychological counselling might be considered as a tool for helping deal with family issues, which is considered as an important correlate for perinatal depression. In Japan, community enhancement involving husbands, parents, and other social organizations, including the working office, companies, and nursing rooms, may be required. In particular, creating a parent-friendly working environment involving colleagues and employers of both the husband and wife, enhancing the social awareness of community members (e.g., family, parents, and colleagues) to have a new consensus that parents who both work and raise a child will be supported in a community, and establishing infrastructure such as nursery rooms, should be prioritized in order to deal with rapid changes in society.

### 4.7. Limitations

Although we included peer-reviewed papers written in English and Japanese and excluded those that had unclear quality, the studies retained in this review had varying statistical effects. However, we included studies that explored factors by means of univariate analysis in order to grasp a wider scope of potential cultural influences on the onset of perinatal depression. Additionally, we did not include Hindi-language articles because we assumed the number of relevant articles is small so far and we cannot assess the quality of these articles. In future studies, updating the findings to include Hindi-language articles may be required to capture broader evidence.

## 5. Conclusions

This literature review synthesized evidence regarding factors relating to perinatal depression in India and Japan. A poor relationship or conflict with the mother-in-law or other family members, and a preference for male infants were identified as specific to India. On the other hand, perinatal depression in Japanese women was characterized by conflict with the biological mother and relatives, concerns about social relationships with other mothers in the community, concerns about childrearing, and conflict with work–life balance. Understanding differences in the social and cultural backgrounds that relate to these factors is important in implementing culture-specific interventions, which may be needed as well as global standardized interventions. In India, involving the family in treatment could be effective, considering that interactions with family members is strongly associated with perinatal depression. In Japan, a community approach involving not only the family but also the working environment is essential for a mother-friendly society.

## Figures and Tables

**Table 1:** Related factors of perinatal depression (India).

Article Number	Author	Language	Study Design	Time Frame	Sample Size	Outcome Measurements	Prevalence (Antenatal)	Prevalence (Postnatal)	Factors of Antenatal Depression	Factors of Postnatal Depression
**I1**	Sheela & Vankatesh (2016) [16]	English	Cross sectional	Between 4–7 weeks postpartum	1600 (analysed: 1600)	EPDS (≥13)	NA	7%	NA	Delayed breastfeeding, female infant
**I2**	Bodhare et al., (2015) [17]	English	Cross sectional	Between 6-8 weeks postpartum	287 (analysed: 274)	PHQ-9	NA	40%	NA	Teenagers, poor education, poor socioeconomic status, depressive symptoms
**I3**	Jain et al., (2015) [18]	English	Cross sectional	Within 2 days postpartum	1537 (analysed: unknown)	EPDS	NA	NA	NA	Mixed breast feeding
**I4**	Shivalli & Gururaj. (2015) [13]	English	Cross sectional	Between 4 and 6 weeks postpartum	118 (analysed: 102)	EPDS (≥13)	NA	65%	NA	Poor socioeconomic status (below poverty line), female baby, complication of pregnancy
**I5**	Srinivasan et al., (2015) [19]	English	Cross sectional	During pregnancy	100 (analysed: 100)	EPDS (≥13)	NA	26%	First trimester, idealistic distortion, partner’s history of depression	NA
**I6**	Johnson et al., (2015) [20]	English	Cross sectional	Within 6-8 weeks postpartum	123 (analysed: 123)	EPDS (≥12)	NA	46%	NA	Depressed mood during pregnancy, staying only with husband, lower self esteem
**I7**	Nongrum et al., (2014) [21]	English	Longitudinal	T1: During pregnancy, T2: After delivery	150 (analysed: 132)	EPDS (≥12)	NA	NA	NA	Domestic violence
**I8**	Lukose et al., (2014) [22]	English	Cross sectional	12 weeks of pregnancy	366 (anallysed: 366)	K-10 ≥ 6	33%	NA	Antenatal depression: Having nausea, vomitting, anemia.	NA
**I9**	Prost et al., (2012) [23]	English	Cross sectional	6 weeks after delivery	5801	K10 scores > 15	NA	12%	NA	Younger age or older age, assetqualities, health problem during pregnancy, health problem during delivery, health problem during postnatal period, alcohol consumption during pregnancy, unwanted pregnancy of father
**I10**	Dubey et al., (2011) [24]	English	Cross sectional	T1: 34 weeks of pregnancy, T2: Within 7 days postpartum	T1: 213 T2: 293 (analysed: 506)	EPDS (≥10)	NA	6%	NA	Family structure, socioeconomic status, marital status, female baby
**I11**	Savarimuthu et al., (2010) [25]	English	Cross sectional	Between 2 and 4 weeks postpartum	137 (analysed: 137)	EPDS (≥12)	NA	26%	NA	Teenagers or older age (>30), education less than 6 years, family history of depression, thought of aborting current pregnancy, unhappy mariage reported, alcohol consumption of husband, delivery of girl
**I12**	Varma et al., (2007) [26]	English	Cross sectional	During pregnancy	203 (analysed: unknown)	BDI	NA	NA	A history of sexual coercion, lower life satisfaction	NA
**I13**	Rodorigues et al., (2003) [27]	English	Qualitative	6–8 weeks postpartum	39 (analysed: unknown)	EPDS (≥12)	NA	NA	NA	Unemployment
**I14**	Patel et al., (2002) [10]	English	Longitudinal	T1: 6-8 weeks postpartum, T2: 6 month postpartum	252 (analysed: 252)	EPDS (≥12)	NA	T1:23% T2: 8%	NA	Antenatal psychiatric morbidity, unplanned pregnancy
**I15**	Chandran et al., (2002) [28]	English	Longitudinal	T1: 34 weeks of pregnancy, T2: 6 weeks after delivery	384 (analysed: 354)	CIS-R (structured interview)	T1:16%	T2:19%	NA	Delivery of female infant, poor support, lower income (<1001 rupees), problems with in laws, poor relationship with parents, nagative life event in previous year

**Table 2:** Factors related to perinatal depression (Japan).

Article Number	Author	Language	Design	Time Frame	Sample Size	Outcome Measurements	Prevalence (Antenatal)	Prevalence (Postnatal)	Factors of Antenatal Depression	Factors of Postnatal Depression
**J1**	Kita et al., (2016) [29]	English	Longitudinal	T1: Late pregnancy T2: One month postpartum	T1: 832 T2: 610 (analysed: 562)	HADS	NA	NA	Antenatal intimate partner violence	NA
**J2**	Iwata et al., (2016) [7]	English	Longitudinal	T1: Within a few days after childbirth T2: One month postpartum	T1–T2: 479 (analysed: 455)	EPDS (≥9)	NA	T1: 21% T2: 21%	NA	Depression early postpartum, financial burden, dissatisfaction with appraisal support, physical burden in daily life, concerns about child rearing
**J3**	Tachibana et al., (2015) [30]	English	Longitudinal	T1: 20 weeks of gestation T2: Within a few days after childbirth T3: One month postpartum	T1: 1717 T2: 1335 T3: 1383 (analysed: 1133)	EPDS (≥9)	NA	T1: 41%	NA	High EPDS score during pregnancy, a perceived lack of family cohesion, primipara, current physical illness treatment
**J4**	Otake et al., (2015) [8]	English	Longitudinal	T1: 25–35 weeks of pregnancy T2: 1–4 months postpartum T3: 6 months postpartum	T1: 309 T2: 267 T3: 154 (analysed: 154)	EPDS (≥9)	T1: 5%	T2: 13%	Past depressive symptoms, worrying, obsessivene character	NA
**J5**	Shirakata et al., (2014) [31]	Japanese	Cross sectional	8–12 weeks, 23–27weeks, and 35–40 gestational weeks of pregnancy	658 (analysed: 352)	CES-D	NA	NA	Severe back pain	NA
**J6**	Fukao & Kabeyama. (2014) [32]	Japanese	Longitudinal	T1: Late pregnancy T2: 2–7 days postpartum T3: One month postpartum	T1–T3: 97 (analysed: 97)	EPDS (≥9)	NA	NA	Lower sense of coherence.	Lower sense of coherence
**J7**	Amagai. (2014) [33]	Japanese	Longitudinal	T1: Late pregnancy T2: One month postpartum	T1: 264 T2: 192 (analysed:153)	EPDS (≥9)	NA	T2: 21%	NA	Financial burden, non-permanent position of partner
**J8**	Minatani et al., (2013) [34]	English	Cross sectional	Late pregnancy	601 (analysed: 601)	EPDS (≥9)	NA	NA	Women’s negative response towards the current pregnancy, low self-directedness and high harm avoidance, perisistence, self transcendence.	NA
**J9**	Kinjo et al., (2013) [35]	Japanese	Cross sectional	T1: During pregnancy T2: One month postpartum	T1: 320 T2: 289 (analysed: 289)	CES-D	T1: 31%	T2: 33%	Unplanned pregnancy, financial burden	Unplanned pregnancy, financial burden, history of depression
**J10**	Sugawara & Ohira. (2013) [36]	Japanese	Longitudinal	T1: Middle pregnancy T2: Late pregnancy T3: One month postpartum	T1–T3: 80 (analysed: 72)	EPDS (≥9)	T1: 16% T2: 12%	T3: 16%	NA	Lower sense of coherence, poor social support
**J11**	Urayama et al., (2013) [37]	Japanese	Longitudinal	T1: Within five days postpartum T2: One month postpartum	T1: 101 T2: 101 (analysed: 100)	EPDS (≥9)	NA	T1: 33% T2: 18%	NA	Lower self efficacy, lower self esteem
**J12**	Sugishita & Kamibeppu. (2013) [38]	Japanese	Longitudinal	T1: Late pregnancy T2: One month postpartum	T1: 161 T2: 121 (analysed:121)	EPDS (≥9)	T1: 14%	T2: 19%	NA	Antenatal depression
**J13**	Miyamoto. (2012) [39]	Japanese	Cross sectional	During pregnancy	128 (analysed: 128)	EPDS (≥9)	15%	NA	Depressive schema, Intrauterine growth restriction (IUGR), poor relationship with partner	NA
**J14**	Nagasaka & Sano. (2012) [40]	Japanese	Longitudinal	T1: Early pregnancy T2: One month postpartum T3: Four month postpartum	T1: 3080 T2: 2420 T3: 2420 (analysed: 2420)	EPDS (≥9)	NA	T2: 13% T3: 12%	NA	T4: teenager, primiparas, unwanted pregnancy, poor support from husband, smoking, history of mental illness, concern about childrearing, economic concern, concern about social relations with other mothers, concern about other relative
**J15**	Hayakawa et al., (2012) [41]	English	Longitudinal	T1: Early postpartum T2: Late pregnancy T3: One month postpartum	T1–T3: 467 (analysed: 448)	EPDS (≥9)	T2: 13.2%	NA	NA	Lower maternal care (PBI)
**J16**	Kokubu et al., (2012) [42]	English	Longitudinal	T1: Late pregnancy T2: Early postpartum T3: One month postpartum	T1–T3: 109 (analysed: 99)	EPDS (≥9)	NA	NA	NA	Antenatal anxiety
**J17**	Kinjo et al., (2011) [43]	Japanese	Cross sectional	T1: During pregnancy T2: One month postpartum	T1: 158 T2: 164 (analysed:164)	CES-D	T1: 30.4%	T2: 24.4%	Perceived stress, lower self esteem, poor social support	Perceived stress, lower self esteem, poor social support
**J18**	Mori et al., (2011) [44]	English	Cross sectional	Within 4 weeks postpartum	675 (analysed: 675)	EPDS (≥9)	NA	T1:11%	NA	Early onset depression (within 4 weeks): Lack of emotional support, psychiatric history Late onset depression (5–12 weeks): younger age(less than 25 years old), older age (older than 36 years old), history of depression
**J19**	Miyake et al., (2011) [45]	English	Cross sectional	Between 3 and 4 months postpartum	771 (analysed: 771)	EPDS (≥9)	NA	14%	NA	Fulltime workers
**J20**	Kikuchi et al., (2010) [46]	Japanese	Longitudinal	T1: Middle pregnancy T2: One month postpartum	T1: 243 T2: 163 (analysed: 113)	EPDS (≥9)	NA	15%	NA	Personality (Neuroticism, low extravert, low agreeableness, low conciousness, emotional oriented coping style), high protection from father
**J21**	Iwamoto et al., (2010) [47]	Japanese	Longitudinal	T1: Late pregnancy T2: 5 days postpartum T3: One month postpartum T4: 4 months postpartum	590 (analysed: 590)	EPDS (≥9)	NA	NA	Unplanned pregnancy	Unplanned pregnancy, pregnancy by infertility treatment
**J22**	Arai & Takahashi. (2009) [48]	Japanese	Cross sectional	One month postpartum	283 (analysed: 149)	EPDS (≥9)	NA	21%	NA	Overall functioning, lower affective responsiveness, lower affective involvement
**J23**	Ando & Muto. (2009) [49]	Japanese	Longitudinal	T1: During pregnancy, T2: 5 weeks postpartum, T3: 3 months postpartum, T4: 6 months postpartum, T5: 1 year after delivery	T1-3: 522 (analysed: 407)	EPDS (≥9)	NA	NA	NA	Unwanted pregnancy, less delighted with fetal movement, morning sickness, conflict with work life balance, poor marital relationship, self absorption, lower self esteem, low attachment with others
**J24**	Satoh et al., (2009) [50]	English	Cross sectional	4 months postpartum	169 (analysed: 169)	EPDS (≥9)	NA	23%	NA	General health abnormality, poor sociability, abnormality, worry about baby care, poor cooperation of the husband
**J25**	Kanazawa et al., (2008) [51]	Japanese	Longitudinal	T1: During pregnancy, T2: Within a few days after delivery, T3: One month postpartum	112 (analysed: 111)	EPDS (≥9)	NA	14%	financial burden, smoking or alcohol comsumption durin pregnancy.	Lower confidence of child rearing, perceived stress with childrearing, concern about baby’s condition
**J26**	Endo et al., (2008) [52]	Japanese	Longitudinal	T1: 5th day postpartum T2: One month postpartum	57 (analysed: 57)	EPDS (≥9)	NA	30%	NA	Older age, maternity blues
**J27**	Mitamura.(2008) [53]	Japanese	Longitudinal	T1: Within a few days after delivery T2: One month postpartum	T1: 549 T2: 503 (analysed: 503)	EPDS (≥9)	NA	8%	NA	Teenagers, divorce during pregnancy or after delivery, history of mental illness, maternity blues
**J28**	Sekizuka et al., (2007) [54]	Japanese	Longitudinal	T1: Late pregnancy, T2: Between 3–5 days after delivery	T1-2: 54 (analysed: 54)	SDS	NA	NA	NA	Lower sense of coherence, lower satisfaction of delivery
**J29**	Sato et al., (2006) [55]	Japanese	Longitudinal	T1: Late pregnancy, T2: 5th day postpartum, T3: One month postpartum, T4: Three month postpartum	T1–T3: 58 (analysed: 58)	SDS	NA	NA	NA	T4: Multipara, Living with extended family, perceived negative relationship with their husband, biological mother, difficulty in baby’s treatment, lack of satisfaction from husband’s support and mother’s support, unbalanced working model, negative feeling toward the baby, negative feeling toword the baby, low maternal attachment, anxiety regarding children
**J30**	Kitamura et al., (2006) [9]	English	Longitudinal	T1: Late pregnancy T2: One month T3: Three months postpartum	T1–T3: 303	SCID	T1: 5%	T2: 5%	Younger age, negative attitude towards the current pregnancy	Poor accomodation, disatisfaction with child sex
**J31**	Ninagawa et al., (2005) [56]	Japanese	Cross sectional	2 month postpartum	332 (analysed: 289)	EPDS (≥9)	NA	16%	NA	Extended family household
**J32**	Suzumiya et al., (2004) [14]	Japanese	Cross sectional	Within 3 months postpartum	3370 (analysed: 3370)	EPDS (≥9)	NA	14%	NA	Pregnancy anomaly, experience of seeing psychiatrist or counsellors, Previous still birth, miscarriage, experience that family has passed away, poor social support from biological parents, poor social support from others, satisfied with regidencial place, financial burden, illness of infant
**J33**	Tokiwa.(2003) [57]	Japanese	Cross sectional	Within seven days pospartum	1500 (analysed: 932)	EPDS (≥9)	NA	NA	NA	Negative birth experience, dissatisfaction with medical staffs, younger age, higher anxiety
**J34**	Iwatani et al., (2001) [58]	Japanese	Longitudinal	T1: Early pregnancy T2: Five days postpartum T3: One month postpartum	T1–T2: 252 (analysed: 252)	SDS (T1 and T2) EPDS (T2)	T1: 31%	T2: 13%	Primiparas	Maternity blues
**J35**	Tamaki et al., (1997) [59]	English	Longitudinal	T1: One month postpartum, T2: Three months postpartum T3: Four months postpartum	T1: 672 T2: 1096 T3: 822 T4: 913 (analysed: 672)	EPDS (≥9)	NA	T1: 18% T2: 12.1% T3: 6.7%	NA	T1: primiparas, worry about child care, higher anxiety, T2: negative life events, worry about childcare, higher anxiety

Note: NA (Not assessed).

## Supplementary Materials

The following are available online at www.mdpi.com/2227-9032/5/4/91/s1. Table S1: Checklist of Inclusion Criteria.
