# Supplementary material for: Cross-National Differences in Psychosocial Factors of Perinatal Depression: A Systematic Review of India and Japan
Source: Healthcare (Basel). 2017 Dec 4;5(4):91. doi: 10.3390/healthcare5040091 (PMC5746725; doi:10.3390/healthcare5040091)
Supplement: Supplementary file 1 [file healthcare-05-00091-s001.pdf]

Table S1. Checklist of Inclusion Criteria.

a.	Study investigates related factors of perinatal depression among Indian or Japanese mothers	Yes ☐ No ☐
b.	Study targeted community dwelling mothers	Yes ☐ No ☐
c.	Study is observational study	Yes ☐ No ☐
d	Study is conducted in India or Japan (not abroad)	Yes ☐ No ☐
e	Study is either quantitative or qualitative design	Yes ☐ No ☐
f	Study is peer reviewed original research	Yes ☐ No ☐
g	Quantitative study meets the minimum quality criteria;	
	g-1. clear description about study design	Yes ☐ No ☐
	g-2. clear description about setting	Yes ☐ No ☐
	g-3. clear description about eligible criterion and the participant numbers	Yes ☐ No ☐
	g-4. clear define all variables (outcomes, exposures, potential confounders.) depression should be assessed by validated measurements.	Yes ☐ No ☐
	g-5. study conducted statistical analysis to identify related factors of perinatal depression.	Yes ☐ No ☐
	g-6. clear description about characteristic of participants and main results (report unadjusted estimate of if applicable confounder-adjusted estimates)	Yes ☐ No ☐
	g-7. study was approved by ethical committee.	Yes ☐ No ☐
